# Investigation of high-dose radiotherapy's effect on brain structure aggravated cognitive impairment and deteriorated patient psychological status in brain tumor treatment

**DOI:** 10.1038/s41598-024-59694-0

**Published:** 2024-05-02

**Authors:** Jianpeng Ma, Hetao Cao, Dongmei Hou, Weiqi Wang, Tingting Liu

**Affiliations:** 1 Department of Magnetic Resonance Imaging, Dingbian County People’s Hospital, Dingbian, Yulin, 718600 Shaanxi China; 2 Department of Medical Imaging, Affiliated Hospital of Nantong University, No.20 Xisi Road, Chongchuan District, Nantong, 226001 Jiangsu China; 3 School of Pharmacy, Nantong University, Nantong, 226019 Jiangsu China

**Keywords:** Radiotherapy, Glioma, Cognitive impairment, Psychological health status, Mini-mental state examination, Neuroscience, Neurology, Oncology, Risk factors

## Abstract

This study aims to investigate the potential impact of high-dose radiotherapy (RT) on brain structure, cognitive impairment, and the psychological status of patients undergoing brain tumor treatment. We recruited and grouped 144 RT-treated patients with brain tumors into the Low dose group (N = 72) and the High dose group (N = 72) according to the RT dose applied. Patient data were collected by using the HADS and QLQ-BN20 system for subsequent analysis and comparison. Our analysis showed no significant correlation between the RT doses and the clinicopathological characteristics. We found that a high dose of RT could aggravate cognitive impairment and deteriorate patient role functioning, indicated by a higher MMSE and worsened role functioning in the High dose group. However, the depression status, social functioning, and global health status were comparable between the High dose group and the Low dose group at Month 0 and Month 1, while being worsened in the High dose group at Month 3, indicating the potential long-term deterioration of depression status in brain tumor patients induced by high-dose RT. By comparing patient data at Month 0, Month 1, Month 3, Month 6, and Month 9 after RT, we found that during RT treatment, RT at a high dose could aggravate cognitive impairment in the short term and lead to worsened patient role functioning, and even deteriorate the overall psychological health status of patients in the long term.

## Introduction

Many patients around the world receive radiotherapy (RT) to treat primary and metastasized tumors in the brain^1,2^. As a fundamental therapy for most brain tumors, RT of the brain is divided into partial brain RT (PBRT) and whole brain RT (WBRT)^3,4^. Currently, more than 100,000 patients in the United States receive brain irradiation each year and more than 50% of these patients display cognitive problems, such as the affected ability of memory, learning, and attention^2,5^. The pathology of cognitive problems caused by radiation is complicated and involves multiple mechanisms. It was proposed that previously unnoticed but subtle indications of irradiation damage to the central nervous system may eventually lead to lasting abnormalities and long-term cognitive disability^6,7^.

The creation of new memory is linked to radiosensitive and mitotically active populations of neural stem cells found in the sub-granular region in the hippocampal dentate gyrus^8^. Damage to these neural stem cells may play a main role in the pathology of early cognitive issues induced by radiation^9^. Pre-clinical studies have also shown that a moderate dose of radiation can trigger a significant reduction in neurogenesis of the sub-granular area^10^. On top of that, the latest medical research has shown a correlation between the dose of radiation and the risk of impaired learning^11^.

Multiple mechanisms have been used to describe the impaired cognitive ability observed after brain RT^12^. Deoxyribonucleic acid damage caused by X-ray radiation may cause cell death and dysfunction. The dynamic cellular process related to oxidative stress and inflammation seems to cause long-term toxicity^13,14^. Moreover, studies have shown the exhaustion of endothelial cells as well as changes to micro-vascularization after brain RT^15^. As an important organ involved in cognition, the hippocampus can be injured during RT and its pathophysiology has been extensively researched^16^. For example, X-ray-induced inhibition of neurogenesis may cause changes in the performance of memory tests, eventually leading to a notable level of cognitive impairment in mice^17–19^. It was also shown that minocycline can prevent neuronal apoptosis induced by radiation during WBI to improve the cognitive function of rats receiving irradiation, clearly demonstrating the protective role of minocycline in neurons against cognitive defects as well as neuronal death induced by radiation^20,21^.

In a previous report on the routine first-line glioblastoma practice, a standard RT treatment did not worsen the cognitive function and psychological status of patients^22^. When observing children with posterior fossa tumors, the verbal comprehension scores were notably decreased in children treated with a higher radiation dose^23^. This study aimed to study the potential influence of high-dose RT on cognitive impairment and patient psychological status in the treatment of brain tumors.

## Materials and methods

### Patient recruitment

A total of 144 brain tumor patients who were subjected to RT were recruited and grouped according to the dosage of RT as the Low dose group (N = 72, patients who received an RT dose no more than 30 Gy) and the High dose group (N = 72, patients who received an RT dose more than 30 Gy). The research was approved in advance by the Ethics Committee of the Affiliated Hospital of Nantong University, and all procedures were carried out in strict compliance with the Declaration of Helsinki. Informed consent was obtained before the initiation of this study. Patient parameters including MMSE, depression, role functioning, social functioning, bladder control, global health status, itchy skin, and weakness of legs were collected from the patients in both groups before the application of RT (Month 0) and at Months 1, 3, 6, and 9 after the application of RT using the HADS and QLQ-BN20 system. All sets of questions were administered to the patients during the baseline assessment, during the visit conducted 2 weeks after the surgical operation, before the start of the concomitant treatment, and during the visits conducted at 1, 3, 6, and 9 months after the start of the concomitant treatment, or when the patients showed progressive disease.

### Patient data collection

For the assessment of cognitive functions and psychological status, the Cognitive Function and Psychological Status Scale, the QLQ-C30 questionnaire, the QLQ-BN20 questionnaire, and the assessment of the European Organization for Research and Treatment of Cancer (EORTC) were used. Furthermore, for the assessment of health-related quality of life (HRQOL), we selected two assessment approaches that were well-established, validated, and most frequently applied in the HRQOL assessment of brain cancer patients: QLQ-BN20 and EORTC QLQ-C30, to assess the status of HRQOL^19,20^. We also utilized the Mini-Mental State Examination (MMSE) to test the general cognitive function of the patients^21^ and the Hospital Anxiety and Depression Scale (HADS) to assess the levels of depression and anxiety of the patients^22^.

As a general multi-dimensional survey frequently applied in the assessment of cancer patients, the EORTC QLQ-C3018 questionnaire contains 30 test items in 5 functional scales (cognitive function, emotional function, physical function, role function, and social function), 3 symptom scales (symptom of fatigue, symptom of nausea and vomiting, and symptom of pain), 1 global health status scale, as well as 6 single item scales to assess additional symptoms like constipation, appetite loss, dyspnea, diarrhea, perceived financial impact, and sleep disturbance. As a questionnaire particularly designed for patients of brain cancer, the EORTC QLQ-BN2019 questionnaire features 20 test items in 11 groups of symptom scales, i.e., motor dysfunction, visual disorders, various disease symptoms such as seizures and headaches, communication deficit, future uncertainty, and treatment toxicities such as hair loss. In this study, the raw score of questionnaires was converted to a linear range ranging from 0 to 100, in which a higher rating indicates a higher functioning level or a greater symptom and problem. Finally, the statistical analysis took into consideration the scores from the six scales of the EORTC QLQ-C30 questionnaire, i.e., physical functioning, cognitive functioning, social functioning, role functioning, emotional functioning, and global health status, as well as all items of the QLQ-BN20 questionnaire.

The short MMSE21 questionnaire of 18 items was used to determine the severity and prognosis of cognitive impairment. In the MMSE scale, a score in the range from 0 to 30 was provided and the lower score represented a more severe level of cognitive impairment. The HADS22 questionnaire including 14 items was used to determine the level of depression and anxiety of the patients. The score of each item in the HADS22 questionnaire ranged from 0 to 3 points, while the overall score in each subscale ranged from 0 to 21 points, and a higher score indicated a higher level of symptomatology.

### Statistical analysis

The statistical analyses of the scores of EORTC QLQ-C30, MMSE, BN20, as well as anxiety and depression were conducted by using mixed effects linear models, to study the improvements or deterioration of cognitive impairment over time between the two groups. In each mixed effects linear model, time analysis was used as a discrete variable, while clinical characteristics as well as the interaction among clinical characteristics for fixed effects were used besides a compound symmetry covariance structure to determine the random effect on the intercept. In addition, the age at the time of diagnosis, gender, and the location of tumors were treated as clinical variables. To compensate for the errors induced by multiple comparisons while minimizing type I errors, *P* = 0.01 was used as the statistical significance level. A difference of ≥ 10 points between the average EORTC scores compared or a difference of ≥ 1.5 between the average HADS scores compared was deemed clinically significant. In addition, patients with an MMSE score of ≤ 26 were deemed to suffer from cognitive function impairment. All statistical analyses were carried out by making use of the SAS version 9.2 statistical software (SAS, Cary, NC).

### Ethics approval

The research was approved in advance by the Ethics Committee of Affiliated Hospital of Nantong University, and all procedures were carried out in strict compliance with the Declaration of Helsinki.

### Consent to participate

Informed consent was received before the initiation of this study.

## Results

### MRI results for brain injury of different patient groups

**Figure 1:**
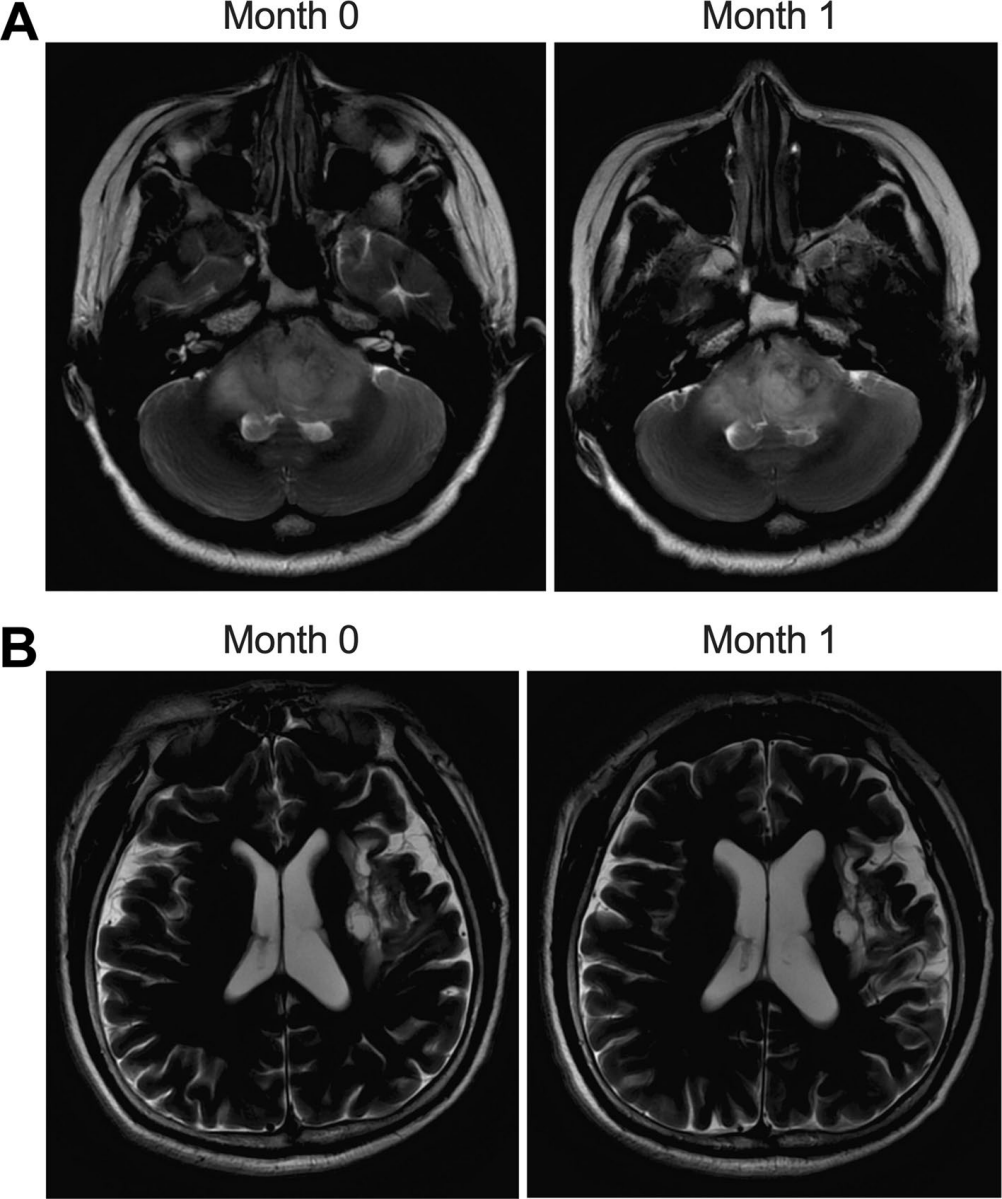
MRI results of brain injury were similar before and after RT. (**A**): Brain injury detected by MRI at Month 0; (**B**): Brain injury detected by MRI at Month 1.

Basic patient clinicopathological characteristics were collected and summarized in Table 1, and no significant differences in these parameters were observed between these two patient groups. Moreover, we also presented the MRI results for brain injury of patients (Fig. 1A) from the Low dose group before the RT (Month 0) and after the RT (Month 1), as well as the MRI results for brain injury of patients from the high dose group (Fig. 1B) before the RT (Month 0) and after the RT (Month 1).

**Table 1:** Basic characteristics of brain tumor patients from the Low dose group and the High dose group

Characteristics	Low dose (N = 72)	High dose (N = 72)	*P* value
Sex, male, n (%)	43 (59.7)	46 (63.9)	0.6087
Age, years	55.8 ± 13.2	51.9 ± 16.7	0.1131
Weight, kg	69.8 ± 15.8	74.1 ± 12.3	0.0705
Height, cm	168.8 ± 11.9	165.4 ± 14.7	0.1294
Right-handed, n	58 (80.6)	53 (73.6)	0.3192
Age at study entry, years	8.5 ± 3.9	9.4 ± 5.1	0.2362
Mean education, years	9.8 ± 3.3	9.3 ± 4.2	0.4283
Mean estimated verbal IQ	95.2 ± 7.5	93.5 ± 8.4	0.2023
Temporal D50%	23.14 ± 12.26	36.32 ± 14.81	< 0.0001
Hippocampus D50%	21.38 ± 11.93	34.52 ± 12.66	< 0.0001
Tumor type			
Low grade glioma	15 (20.8)	12 (16.7)	0.5300
High grade glioma	25 (34.7)	27 (37.5)	0.7275
Primary CNS lymphoma	26 (36.1)	23 (31.9)	0.5960
Others	6 (8.4)	10 (13.9)	0.2961
Tumor location			
Frontal/frontal–temporal/frontal-parietal	35 (48.6)	38 (52.8)	0.6155
Temporal/parietal/occipital	16 (22.2)	20 (27.8)	0.4394
Cortical/subcortical	21 (29.2)	14 (19.4)	0.1719
Predominant tumor side			
Left	21 (29.2)	21 (29.2)	1.0000
Right	26 (36.1)	27 (37.5)	0.8622
Bilateral	25 (34.7)	24(33.3)	0.8597
Radiation field			
Craniospinal + boost	27 (37.5)	31 (43.1)	0.4948
Focal	45 (62.5)	41 (56.9)	0.4948
KPS at baseline			
100	16 (22.2)	15 (20.8)	0.8385
90	5 (6.9)	6 (8.4)	0.7358
80	35 (48.6)	38 (52.8)	0.6155
70	16 (22.2)	13 (18.0)	0.5309

D50%: median dose, the dose of 50% volume of PTV (planning total volume).

### High-dose RT deteriorates cognitive impairment in the short term

**Figure 2:**
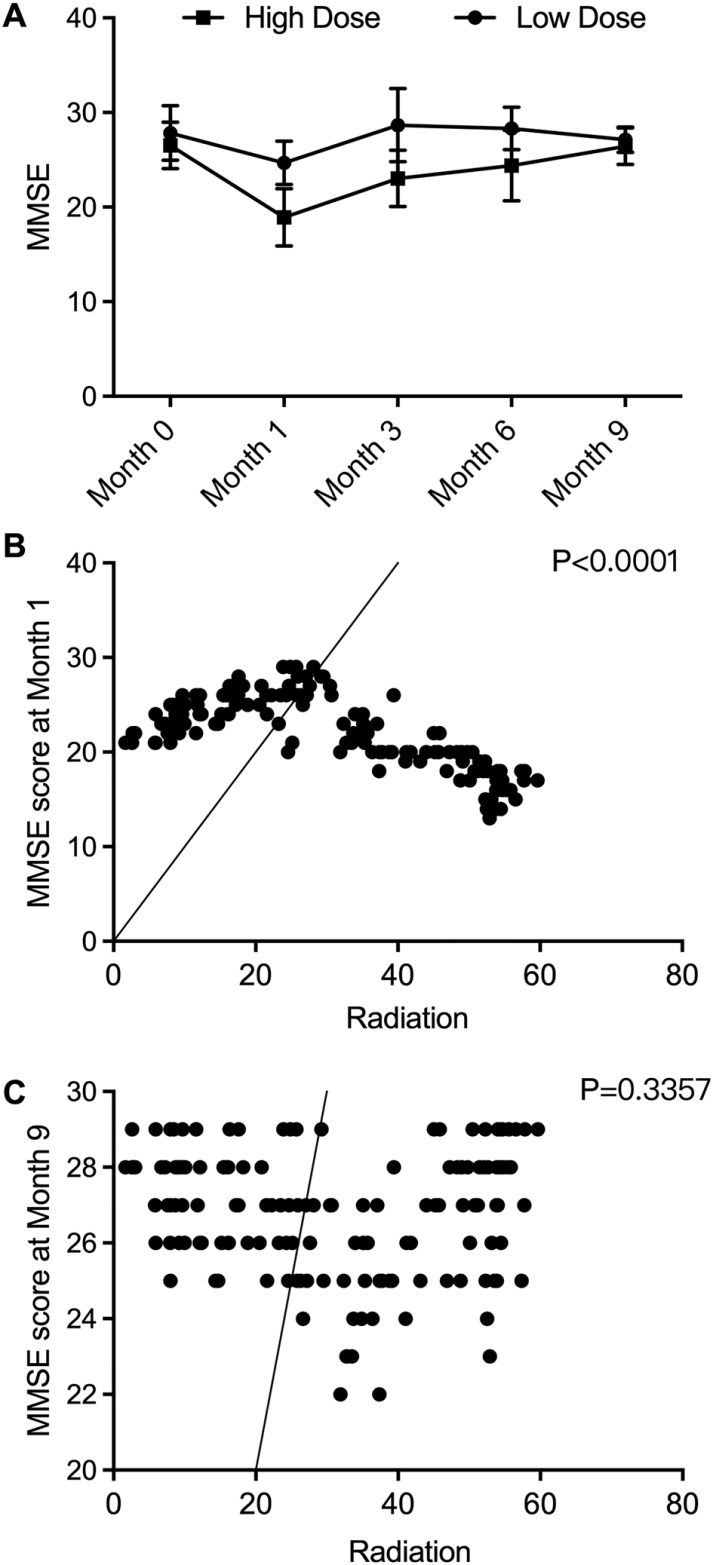
High-dose RT aggravated cognitive impairment in the short term. and aggravated depression in the long term (* P value < 0.05 compared with low dose group). (**A**): MMSE score of High dose group and Low dose group recorded before RT (Month 0), Month 1, Month 3, Month 6 and Month 9 indicated acute adverse effect of high-dose RT on cognitive impairment; (**B**): Correlation analysis between radiation dose and MMSE score at Month 1; (**C**): Correlation analysis between radiation dose and MMSE score at Month 9.

To predict the effect of different doses of RT on patient neurocognitive status, the MMSE score system was utilized. As shown in Fig. 2A, the cognitive impairment of the Low dose and High dose groups was similar before the treatment of RT (Month 0). However, at Months 1, 3, 6, and 9 after the treatment of RT, a significant reduction in the MMSE scores was observed in patients treated with high-dose RT compared with those treated with low-dose RT, indicating the potential acute negative effect of the higher dose of RT on patient cognitive impairment. Moreover, when analyzing the possible correlation between radiation dose and individual patient’s MMSE scores recorded at Month 1 or Month 9 after RT (Fig. 2B), we found a significant negative correlation between the high dose of radiation and cognitive impairment after the first month of RT, while no significant correlation was demonstrated between radiation dose and cognitive performance after the ninth month of RT (Fig. 2C). Therefore, it is suggested that high-dose RT may exhibit an acute adverse effect on cognitive performance.

### High-dose RT aggravates depression, deteriorates patient role functioning, and obstructs social functioning in the long term

**Figure 3:**
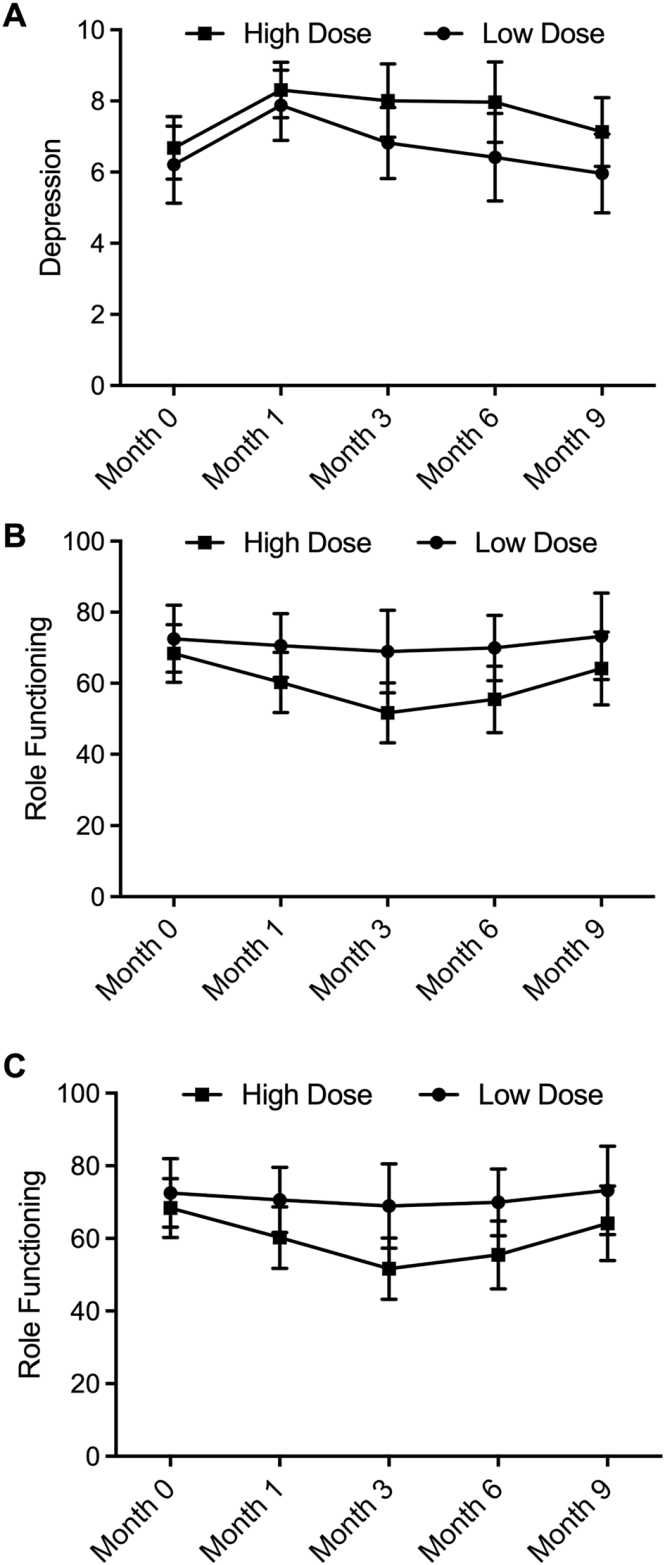
High-dose RT aggravated depression, deteriorated patient role functioning and obstructed social functioning in the long term (* P value < 0.05 compared with low dose group). (**A**): Depression score of the High dose and the Low dose group recorded before RT (Month 0), Month 1, Month 3, Month 6 and Month 9 indicated long-term adverse effect of high-dose RT on depression; (**B**): The role functioning was deteriorated by high-dose RT in the long term according to role functioning score recorded before RT (Month 0), Month 1, Month 3, Month 6 and Month 9; (**C**): The social functioning was obstructed by high-dose RT in the long term according to role functioning score recorded before RT (Month 0), Month 1, Month 3, Month 6 and Month 9.

Meanwhile, as indicated by Fig. 3A, depression status, as observed by HADS between the High dose group and the Low dose group, showed interesting patterns. The differences between the Low dose and High dose groups were insignificant at Months 0, 3, 6, and 9, while the depression status was worsened in the High dose group compared with the Low dose group at Month 1, indicating the potential long-term deterioration of depression status of patients induced by high-dose RT.

As shown in Fig. 3B, the role functioning of both patient groups measured by QLQ-BN20 showed a similar tendency as the MMSE scores. At Month 0, the role functioning was comparable in both patient groups, while the patients in the High dose group showed a lower role functioning score at Months 1, 3, 6, and 9, indicating that high-dose RT could deteriorate the role functioning of brain tumor patients in the long term.

Unlike role functioning, social functioning (Fig. 3C) measured by QLQ-BN20 was similar between the Low dose group and the High dose group before RT and at 1 month after RT. However, since the third month after RT, the social functioning of patients receiving high-dose RT decreased compared with that of patients receiving low-dose RT, thus indicating that high-dose RT could obstruct patient social functioning in the long term.

### High-dose RT deteriorated global health status in the long term while exhibiting no effect on patient bladder control, itchy skin, and weakness of legs

**Figure 4:**
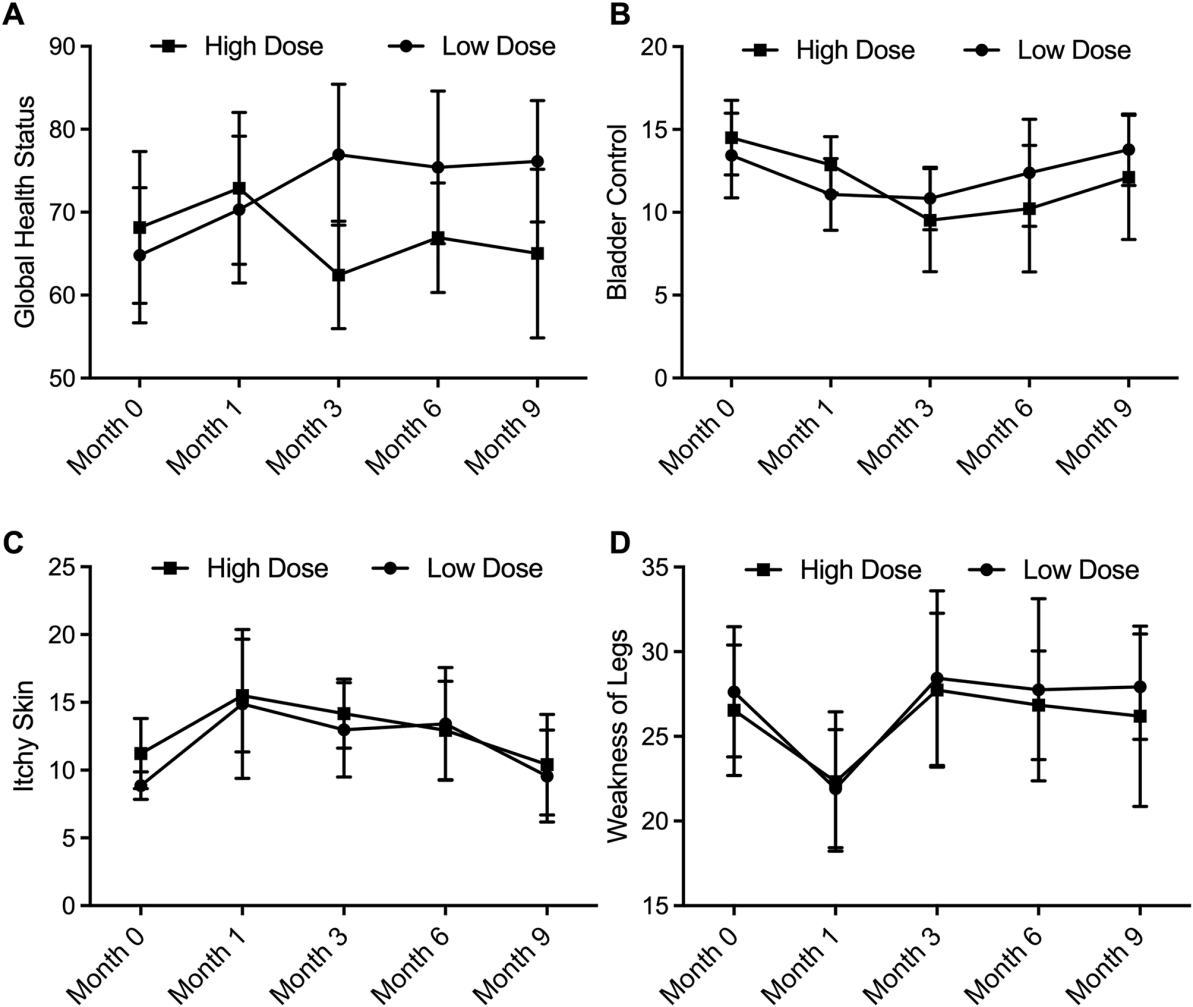
High-dose RT deteriorated global health status in the long term, while RT exhibited no effect on patient bladder control, itchy skin or weakness of legs (* P value < 0.05 compared with low dose group). (**A**): Patient global health status was comparable between the the Low dose group and the High dose group at Month 0 and Month 1, while being deteriorated in the High dose group since Month 3; (**B**): Patient bladder control conditions were comparable between the Low dose group and the High dose group at Month 0, Month 1, Month 3, Month 6 and Month 9; (**C**): Patient itchy skin conditions were comparable between the Low dose group and the High dose group at Month 0, Month 1, Month 3, Month 6 and Month 9; (**D**): Patient weakness of legs status was comparable between the Low dose group and the High dose group at Month 0, Month 1, Month 3, Month 6 and Month 9.

As shown in Fig. 4A, the global health status of brain tumor patients was recorded before and after RT. At Months 0 and 1, the global health status of the Low dose group and the High dose group was comparable. However, since the third month after RT, the score for global health status decreased in patients receiving high-dose RT. Meanwhile, other parameters including patient bladder control, itchy skin, and weakness of legs were compared between the Low dose group and the High dose group before RT (Month 0) and at Months 1, 3, 6, and 9 after RT. As indicated by the results, no statistical significance was found between patients treated with high-dose RT and low-dose RT concerning patient bladder control (Fig. 4B), itchy skin (Fig. 4C), or weakness of legs (Fig. 4D) at all time points, thus demonstrating that the dose of RT was not statistically correlated with itchy skin and weakness of legs.

## Discussion

While effective in treating cancer, radiotherapy (RT) also exerts side effects, such as cognitive impairment. Factors including the location of RT treatment, the dosage and duration of RT, the age and health status of patients receiving RT, and even the types of radiation may influence the onset of its side effects^24^. For radiation directed toward the brain or surrounding tissue areas, the risk of cognitive impairment is increased. Also, higher doses and longer durations of RT treatment can increase the risk of cognitive side effects. Aged adults with existing cognitive conditions were also found to be more susceptible to cognitive impairment as a side effect of RT treatment^25^. Proton therapy, compared with other types of radiation, is associated with a lower risk of cognitive impairment due to its greater precision in sparing surrounding healthy tissues^26^. In this study, we recruited brain tumor patients who received RT. By comparing the MRI results before RT and 1 month after RT, we found no significant structural changes. However, significant effects on cognitive functions were observed. After RT, the MMSE scores of the High dose group were lower than those of the Low dose group at Month 1 and Month 3, indicating that a higher dose of RT could have a negative effect on the patients' cognitive functions. Role functioning exhibited a similar tendency as that of the MMSE scores between the High dose group and the Low dose group at Months 0, 1, 3, 6, and 9, demonstrating that RT at a higher dose could deteriorate the role functioning of brain tumor patients. In addition, the depression status measured by HADS, as well as the social functioning and global health status measured by QLQ-BN20, were comparable between the High dose group and the Low dose group at Months 0 and 1. Meanwhile, the depression status, social functioning, and global health status all worsened in the High dose group at Month 3, indicating the potential long-term deteriorative effect of high-dose RT on the depression status of brain tumor patients.

Since brain tumors may result in isolated deficiencies, global scores of patient functionality may not show clinically crucial prognostic information about the patients if the deficiencies in motor or cognitive domains have various implications for survival. It was shown that neuropsychological examinations had a prognostic value in pooled samples of patients with glioblastoma and recurring anaplastic astrocytoma^27,28^. Various other studies also assessed the link between Mini-Mental Condition Examination (MMSE) scores and patient survival, and some revealed that MMSE impairment is linked with much shorter survival in individuals with newly diagnosed glioblastoma or low-grade glioma^29,30^.

A previous study showed that the apoptosis of neurons in the hippocampus in rodent brain tissues may cause delayed issues of cognitive deficits after RT^31–33^. Another research also showed that the number of viable neurons in the hippocampal CA1 domain was reduced after WBRT. In this study, it is noteworthy that the hippocampus D50% parameter was significantly higher in the High dose group (34.52 ± 12.66) compared with the Low dose group (21.38 ± 11.93). As a critical structure within the brain's medial temporal lobe, the hippocampus plays a critical role in memory formation, organization, and retrieval, as well as in spatial navigation. Conditions such as Alzheimer's disease, epilepsy, and depression are associated with hippocampal dysfunction, leading to memory deficits and other cognitive impairments. The impacts of hippocampal dysfunction on cognitive functions have been extensively studied in previous investigations^34,35^. Since the hippocampus D50% was higher for the High dose group, the hippocampal function may be potentially influenced by RT treatment as well. However, parameters including bladder control, itchy skin, and weakness of legs were not influenced by the dose of RT. The bladder control, itchy skin, and weakness of legs in the Low dose group were similar to those in the High dose group at Month 0, Month 1, Month 3, Month 6, and Month 9, demonstrating that the dose of RT was not statistically correlated with bladder control, itchy skin and weakness of legs.

Microglia and astrocytes can react to the irradiation of the brain by producing certain factors to cause neuro-inflammation as well as influence the differentiation and function of cells in the CNS^5,6^. Pro-inflammatory cytokines including tumor death factor-α, interleukin-1β, interleukin-6 as well as interleukin-18 were assayed in the brain's particular regions after radiation^14^. Inflammatory biomarkers like GFAP, NF-κb, as well as intercellular adhesion molecule-1 were additionally characterized in brain tissues after radiation^36^.

Apart from the extent of resection, the differences in functioning levels between different patient groups may also be attributed to other key factors. It has been demonstrated that there was a correlation between the subtotal resection procedure and the worse baseline functioning^37^. In another previous study on nasopharyngeal carcinoma patients, the correlation between cognitive features, lesion volume, and lesion site was identified. A strong correlation was shown between the site of radio necrosis and the forms of cognitive impairment^38^. Currently, the combination of RT and surgical treatment is the approach offering significant advantages of survival^39,40^. Temozolomide has also been added to RT to present a substantial advantage in OS and progression-free survival^41^. Most of the above studies showed a detrimental impact of RT on cognitive performance. The tests of executive function, memory, and motor coordination also showed a correlation between tumor volume and cognitive performance.

However, the findings of this study must be interpreted within the context of several limitations. First, the relatively small sample size could limit the statistical power of our analysis, suggesting that future studies with larger cohorts are necessary to validate our findings. Additionally, the lack of chemotherapy information and comorbidities from our patients’ data represents a significant limitation as these factors may influence cognitive functions or interact with RT to exacerbate cognitive decline, thereby confounding our results. Future research should aim to incorporate these parameters to provide a more comprehensive understanding of the multifactorial nature of cognitive impairment in brain tumor patients. Thirdly, the absence of information regarding the brain areas and volumes that received RT treatment further complicates the interpretation of our results, limiting the precision of our conclusions. Moreover, the lack of resection extent in each patient group also raises questions about the potential confounding effects of surgical variables on our findings, since this factor combined with significant differences in hippocampal dose between the two groups may suggest that our data may not solely reflect the impact of radiotherapy dose but also the influence of surgical outcomes and individual patient differences. With respect to these limitations, our study highlights the need for further research that employs larger, more diverse patient cohorts, including comprehensive treatment and comorbidity data, and detailed dosimetry analysis.

## Conclusion

In conclusion, by measuring the cognitive impairment and other psychological health-related parameters at Month 0, Month 1, Month 3, Month 6, and Month 9 after RT, we found that during the treatment of brain tumor, RT at a high dose could aggravate cognitive impairment in the short term and lead to worsened patient role functioning, and even deteriorate the overall psychological health status of patients in the long term.

## Data Availability

The data that support the findings of this study are available from the corresponding author upon reasonable request.

## Abbreviations

PBRT: Partial brain radiotherapy
WBRT: Whole brain radiotherapy
KPS: Karnofsky performance status
MMSE: Mini-mental state examination
HADS: Hospital anxiety and depression scale
EORTC: European organization for research and treatment of cancer
HRQOL: Health-related quality of life
